# Glutamic‐acid grafted hyaluronic acid inhibits inflammatory factors via fibroblast and skin model tests

**DOI:** 10.1111/srt.13548

**Published:** 2024-01-04

**Authors:** Fang‐ru Jiang, Song Wang, Dong Han, Jian Wei, Ya‐nan Wu, Zhe Liu

**Affiliations:** ^1^ Bloomage Biotech Co., Ltd Jinan Shandong China; ^2^ Department of Dermatology the Third People's Hospital of Hangzhou Hangzhou Zhejiang China

**Keywords:** glutamic acid (Glu), hyaluronic acid (HA), inflammation

## Abstract

**Background:**

Excessive inflammation may cause tissue damage and disrupt the function of the skin barrier. Hyaluronic acid (HA), an endogenous component, was found to regulate multiple inflammatory factors for skin health. This work aims to further enhance its efficacy by grafting amino acid onto its molecule.

**Methods:**

Glutamic acid (Glu) was selected as the ligand to react with low‐molecular‐weight HA. Fibroblast tests and a 3D skin model were used to investigate the anti‐inflammation efficacy of HA‐Glu.

**Results:**

For IL‐1α, IL‐6 and TNF‐α, the grafted compound presents stronger inhibition ability versus native HA. Moreover, HA‐Glu could promote the repair of damaged skin by improving the compactness of the stratum corneum and increasing the thickness of the living cell layer.

**Conclusion:**

The application of HA‐Glu compound in skin care formulas would be effective to alleviate inflammation‐induced skin symptoms and skin aging.

## 1 INTRODUCTION

Inflammation is a natural immune response to infection, injury, or other harmful stimuli, which promotes tissue repair and regeneration. However, chronic, or excessive inflammation would cause tissue damage and disrupt skin barrier function, leading to various skin problems, such as aging, acne, eczema, and psoriasis.^1^

Interleukin‐1 alpha (IL‐1α), Interleukin‐6 (IL‐6), and Tumor necrosis factor‐alpha (TNF‐α) are three typical factors participating in multiple inflammatory processes.^2^, ^3^ Bou‐Gragham's study showed that overexpression of IL‐1α was associated with the worsening of symptoms and disease progression in conditions such as atopic dermatitis, neutrophilic dermatoses, skin phototoxicity, and skin cancer.^4^ Similarly, IL‐6 was found to activate TGF‐β, NF‐κB, STAT3, and ERK pathways, while also triggering oxidative stress through the production of reactive oxygen species.^5^ In Khan's research, the elevated IL‐6 level was found to correlate with the severity of skin manifestations and the degree of skin thickening, both of which contribute to skin fibrosis—a characteristic feature of systemic sclerosis.^6^ Additionally, Firlej's research disclosed that excessive TNF‐α production can recruit immune cells, release additional inflammatory mediators, and result in tissue damage, as well as contributing to acne scarring.^7^ Also, TNF‐α could induce ROS and activate both activator protein 1 (AP‐1) and nuclear factor‐kappa B, leading to the generation of interstitial collagenase (MMP‐1) and the subsequent degradation of collagen fibrils.^8^, ^9^

In order to suppress excessive skin inflammation, various ingredients were studied, and one of them is hyaluronic acid (HA), which was found to regulate multiple inflammatory factors. In Rayahin^10^ and Zheng's^11^ researches, low‐molecular‐weight HA (LMWHA) was found to regulate the expression of nos2, TNF‐α and cd80, and to reduce the expression of IFN‐γ and IL‐4. Meanwhile, Zheng^11^ indicated that LMWHA could decrease the expression of NO and IL‐6.^12^ discovered that LMWHA could significantly decrease the production of IL‐18 in human keratinocytes induced by contact allergens like 2,4‐dinitrochlorobenzene (DNCB) and PPD. In addition, Chen^13^ et al. elucidated the multiplicity of mechanisms through which a newly discovered Hyaluronic Acid (HA) complex counters intrinsic skin aging. In typical dermal fibroblasts, a blend of Hyaluronic Acid complexes constituted of low molecular weight Sodium Hyaluronate (30 kDa) and its acetylated derivatives exhibited a synergistic effect in the inhibition of Matrix Metalloproteinase‐1 (MMP‐1) expression. Concurrently, these complexes stimulated an accumulation of Type I collagen and the expression of dermo‐epidermal junction proteins, thereby demonstrating anti‐aging effects on skin in vitro.

This work aims to further enhance the anti‐inflammation property of LMWHA via grafting glutamic acid (Glu) onto it. Since L‐glutamate (Glu) was reported to regulate inflammation, such as modulating the release of IL‐6,^14^ and interacting with mGlu5R,^15^ the grafted molecule is expected to deliver stronger efficacy than native HA. To demonstrate the benefits, both cell experiments and a 3D skin model were used in the study.

## 2 MATERIALS AND METHODS

### 2.1 Synthesis of HA‐Glu

Sodium Hyaluronate (HA‐Na) with the average molecular weight of 30 kDa was employed for the reaction, which was supplied by Bloomage Biotechnology Co., Ltd. (China) with >99% purity. The other chemicals, that is, hydrochloric acid (HCl), tetrabutylammonium hydroxide (TBA‐OH), N, N‐Dimethylformamide (DMF), 2‐chloro‐1‐methylpyridinium iodide (CMPI), L‐glutamic acid diethyl ester hydrochloride (Glu‐HCl), triethylamine (TEA), sodium hydroxide (NaOH), and Sodium chloride (NaCl) were analytical grade reagents with >99% purity purchased from Shanghai Macklin Biochemical CO., LTD. (China).

**FIGURE 1:**
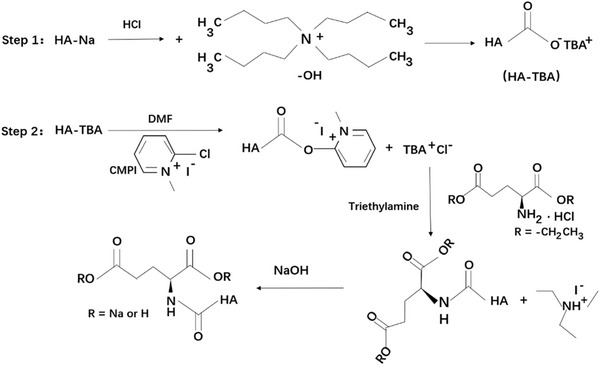
Synthesis reaction of HA‐Glu.

The synthesis method was adapted from Carole^16^ and Magnani's^17^ reports, whose pathway was described as 2 steps, shown in Figure 1. In Step 1, HA‐Na was dissolved in ethanol and acidified by HCl. After filtration, a solid residue was obtained. The residue was then dissolved in water and reacts with TBA‐OH to form HA‐TBA solutions. Further, the solution was dried to obtain the solid HA‐TBA.

In Step 2, HA‐TBA was dissolved in DMF, followed by the addition of CMPI, Glu‐HCl and TEA for overnight mixing at room temperature. After this, DI water and NaOH were dosed to initiate hydrolysis, and then, the pH value was appropriately adjusted to 6.5 by using HCl. NaCl was then added to the solution. Finally, purification was performed with anhydrous ethanol and followed by filtration. The resulting solid was dried to obtain HA‐Glu.

Throughout the chemical grafting process, the stereoscopic property of L‐glutamate was maintained. Firstly, the breaking of C‐N bond was avoided in the reaction pathways. Secondly, stringent control was exercised over the pH levels during all stages, ensuring they stayed within the 5–9 range. Furthermore, the temperature during the reaction and drying stages was also carefully regulated to remain below 40°C, thereby preventing any extreme conditions.

By adjusting the dosage levels of HA‐Na, Glu‐HCl and TEA, different degrees of grafting can be achieved. In Table 1, the reaction formulas and grafting degrees were presented.​

**TABLE 1:** Detailed formulas of different graft degree HA‐Glus.

Code	HA‐Na (mmol)	Glu‐HCl (mmol)	TEA (mmol)	Relative proton ratio	grafting rate (%)
Graft‐1	1	0.3	1.5	0.27	27
Graft‐2	1	0.6	1.7	0.47	47
Graft‐3	1	1.3	2.5	0.87	87

### 2.2 Cell experiments

Human dermal fibroblasts (HDFs, lot# Fb22113007) were employed for cell tests. DMEM basic(1X) medium (DMEM, Cat# C11995500BT, Thermo), fetal bovine serum (FBS, Cat# 10091148, Thermo), and penicillin‐streptomycin solution (PS, Cat# 15140122, Thermo), sourced from Zhejiang Hunter Biotech Company (China), were used as the supporting materials.

The test design was shown in Table 2: the native HA and Glu compounds were compared to the grafted molecules at the identical concentration. Blank, negative, and positive controls (Cell‐BC, ‐NC, and ‐PC) were included as the references.

**TABLE 2:** Experimental design of cell tests.

Case	Test formulas
Cell‐BC	blank control
Cell‐NC	negative control
Cell‐PC	129 ng/mL Dexamethasone sodium phosphate
Cell‐1	150 μg/mL HA
Cell‐2	150 μg/mL Glu
Cell‐3	150 μg/mL Graft‐1
Cell‐4	150 μg/mL Graft‐2
Cell‐5	150 μg/mL Graft‐3

For testing, the culture medium was first prepared by mixing DEME, FBS and PS at the ratio of 89:10:1. Then, HDFs were cultured in the medium for 24 h under the condition of 37°C, 5% CO_2_. After that, the cells were exposed to a 320 nm wavelength UV light with an energy of 80 mJ/cm^2^ for 60 s and then treated by the formulas. After another 24 h of culture, the measurements were performed. Gene expressions of IL‐1α, TNF‐α, and IL‐6 were quantified by a real‐time reverse transcription PCR (qRT‐PCR) assay. To be noted, the blank control (Cell‐BC) was not exposed to UV or formulas, and the negative control (Cell‐NC) was only exposed to the UV light but not treated by formulas.

### 2.3 3D skin experiments

3D skin samples (EpiKutis, lot# ES230403) and culture medium (EpiGrowth, Cat. # PY3021), supplied by Guangdong Biocell Biotechnology Co., Ltd (China), were employed. The following chemicals from Sigma‐Aldrich were used as the supporting materials: sodium lauryl sulfate (SLS, Cat. # 1614363), dexamethasone (Cat. # D4902), pirinixic acid (Wy‐14643, Cat. # C7081), and phosphate‐buffered saline (PBS, Cat. # 806552). In addition, Human IL‐6 ELISA Kit (ab178013, Abcam), Human TNF‐alpha ELISA Kit (ab285312, Abcam) and Human IL‐1 alpha ELISA Kit (ab100560, Abcam) were used for the enzyme‐linked immunosorbent assay (ELISA) test. Hematoxylin and Eosin Staining Kit (Cat. # C0105S), from Beyotime company, was utilized to stain skin slices.

In the experiments, native HA (Model‐1) was tested versus the mixture of two grafted HA‐Glu compounds (Model‐2). The mixture contains Graft‐1 and Graft‐3 with a 1:1 weight ratio. As shown in Table 3, blank, negative, and positive controls were included for reference.

**TABLE 3:** Experimental design of 3D skin model tests.

Case	Test formulas
Model‐BC	blank control
Model‐NC	negative contro
Model‐PC1	16.19 μg/mL Pirinixic acid
Model‐PC_2_	100 μg/mL Dexamethasone
Model‐1	500 μg/mL HA
Model‐2	250 μg/mL Graft‐1, 250 μg/mL Graft‐3

Skin samples were placed in a 6‐well plate with 0.9 mL EpiGrowth^®^ medium. With the exception of Model‐BC, all samples were irritated by a 25 μL 0.1% SLS solution for 30 min to cause skin damage and inflammation. The skin surface irritants were then rinsed by PBS and wiped by a sterile cotton swab. After that, the formulas were dosed and the samples were cultured for 24 h under the condition of 37°C, 5% CO_2_. The ELISA test was conducted using the microplate reader (BioTek, Epoch). At the same time, cross sections of skin samples were observed and analyzed.

### 2.4 Statistical analysis

The mean value and standard deviation of each case were calculated from 6 replicates. one‐way ANOVA was used to analyze the differences between multiple groups. If the results of one‐way ANOVA indicated significant differences, further comparisons between the two groups were conducted as needed. For multiple comparisons between the two groups, Bonferroni method was used to correct the *p*‐value. A *p* value <0.05 was considered statistically significant.

## 3 | RESULTS

### 3.1 Molecular structure

**FIGURE 2:**
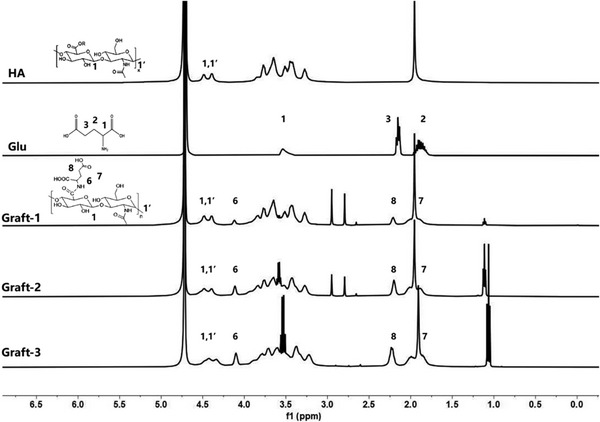
^1^H NMR spectra of native HA, L‐Glu, and HA‐Glus.

The structures of HA‐Glu molecules were verified by NMR (BRUKER, Avance III HD 500 MHz), and the ^1^H NMR spectra were presented in Figure 2. For comparison, the spectra of HA and Glu were also plotted, with hydrogen atoms at distinct positions being denoted by varying numbers. It can be observed that the chemical shifts of the methylidyne group (‐CH‐, 1) and methylene group (‐CH_2_‐, 3) in Glu molecule are *δ* = 3.53 ppm and *δ* = 2.15 ppm, respectively. While, in HA‐Glu molecule the shift of 1 (derived from Glu) moves to the low field with *δ* = 4.09 ppm (6), indicating that Glu was grafted onto HA. Concurrently, the shift of 3 (derived from Glu) moves to 8 (*δ* = 2.19–2.22 ppm).

The grafting degree can be calculated as follows: In ^1^H NMR spectra, the absorption peaks of HA appearing at *δ* = 4.37 and 4.46 ppm (1,1′) were used as the standard, with the integrated proton ratio denoted as A. These peaks represent the absorption of one proton in the glucuronic acid structural unit and another proton in the N‐acetylglucosamine structural unit of the HA molecule. For HA‐Glu molecule, the absorption peak appearing at *δ* = 4.09 ppm (6) has an integrated proton ratio, denoted as B. This peak represents the absorption of the methylidyne group (‐CH‐) in Glu ligand of the grafted compound. Then, the grafting degree can be calculated as B/(A/2) × 100% (Figure 2).

### 3.2 Cell test

**FIGURE 3:**
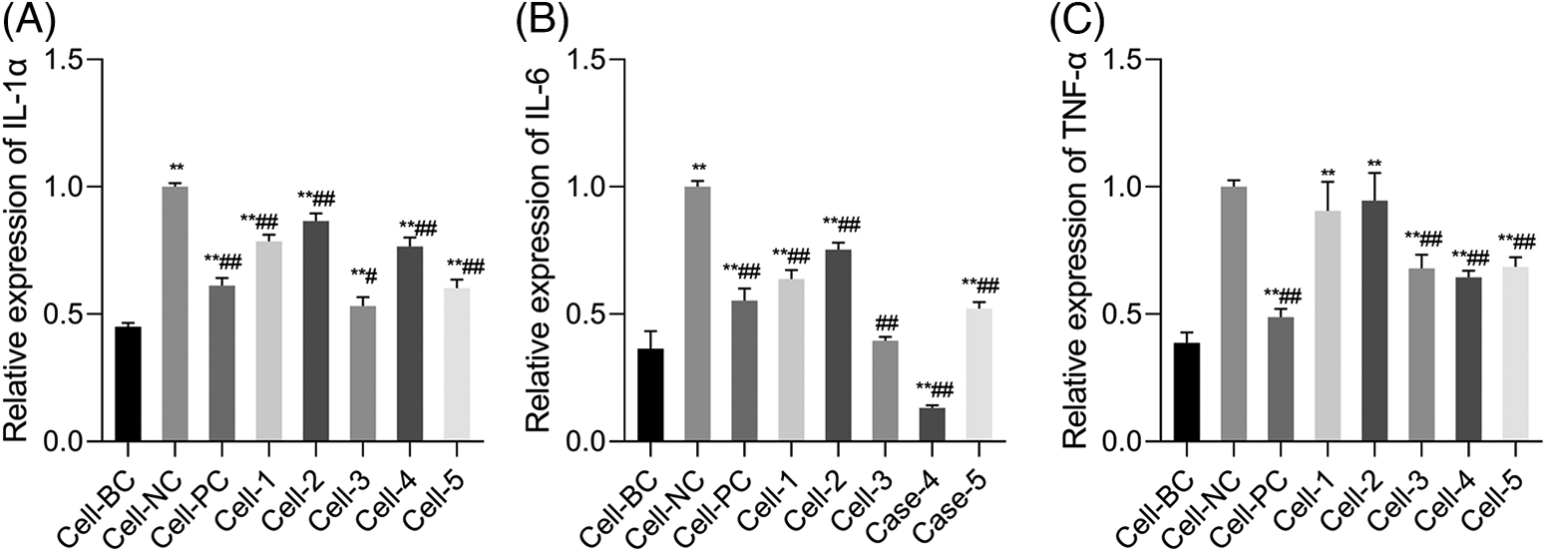
The expression levels of IL‐1α, IL‐6 and TNF‐α in cell tests were detected by qRT‐PCR. Results of IL‐1α (A), IL‐6 (B), and TNF‐α (C) expression in cell tests. (Mean ± SD, *n* = 6). Cell‐BC: blank control, Cell‐NC: negative control, Cell‐PC: 129 ng/mL Dexamethasone sodium phosphate, Cell‐1: 150 μg/mL HA, Cell‐2: 150 μg/mL Glu, Cell‐3:150 μg/mL Graft‐1, Cell‐4: 150 μg/mL Graft‐2, Cell‐5: 150 μg/mL Graft‐3. **p < 0.01 versus Cell‐BC; ##*p* < 0.01 versus Cell‐NC.

The gene expressions of IL‐1α, IL‐6, and TNF‐α were summarized in Figure 3. It can be seen that native HA (Cell‐1) could significantly inhibit the expression of IL‐1α and IL‐6 compared with the negative control (Figure 3A,B), which is consistent with Zheng's learning.^11^ Meanwhile, Glu (Cell‐2) also presents the ability to decrease the levels of inflammatory factors, but the efficacy is weaker than that of HA. However, neither HA nor Glu could suppress the expression of TNF‐α versus negative control (Figure 3C).

The superiority of the grafted molecules can be observed by comparing the results obtained from Cells 1–2 versus those obtained from Cells 3–5. At the given concentration, majority of HA‐Glu compounds exhibit stronger inhibitory effects than native HA and Glu. Regarding IL‐1α, HA‐Glu molecules with low and high grafting degrees (Cell‐3 and 5) show advantages over HA and Glu, but the results of the medium‐grafted molecule (Cell‐4) do not differ significantly from those of HA. For IL‐6, all three grafted molecules showed strong inhibition, with the medium grafted molecule performing the best. Notably, although HA and Glu show no inhibitory impact on TNF‐α expression, their grafting products exhibit such an effect. Additionally, no significant difference was observed in TNF‐α suppression among HA‐Glu molecules, regardless of the grafting degree (Figure 3).

### 3.3 3D skin model

**FIGURE 4:**
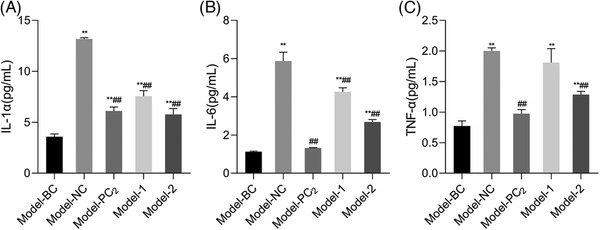
The expression levels of IL‐1α, IL‐6 and TNF‐α in 3D model tests were detected by ELISA. Results of IL‐1α (A), IL‐6 (B), and TNF‐α (C) expression in 3D model tests. (Mean ± SD, *n* = 6). Model‐BC: blank control, Model‐NC: negative control, Model‐PC_1_: 16.19 μg/mL Pirinixic acid, Model‐PC_2_: 100 μg/mL Dexamethasone, Model‐1: 500 μg/mL HA, Model‐2: 250 μg/mL Graft‐1, 250 μg/mL Graft‐3. **p < 0.01 versus Model‐BC; ##*p* < 0.01 versus Model‐NC.

The anti‐inflammation effect of HA‐Glu was further demonstrated by a 3D skin model. Since low‐ and high‐grafted molecules present excellent inhibition in all three factors, their mixture with 1:1 weight ratio was employed as the test leg to compare with native HA. Figure 4 showed the results of IL‐α, IL‐6, and TNF‐α expressions. One can find that the inhibition efficacy is consistent with cytological outcomes, where both native HA and HA‐Glu mixture deliver anti‐inflammation effects, and the ratio of function enhancement of HA‐Glu over native HA is similar to what observed in cellular assays (Figure 4)

**FIGURE 5:**
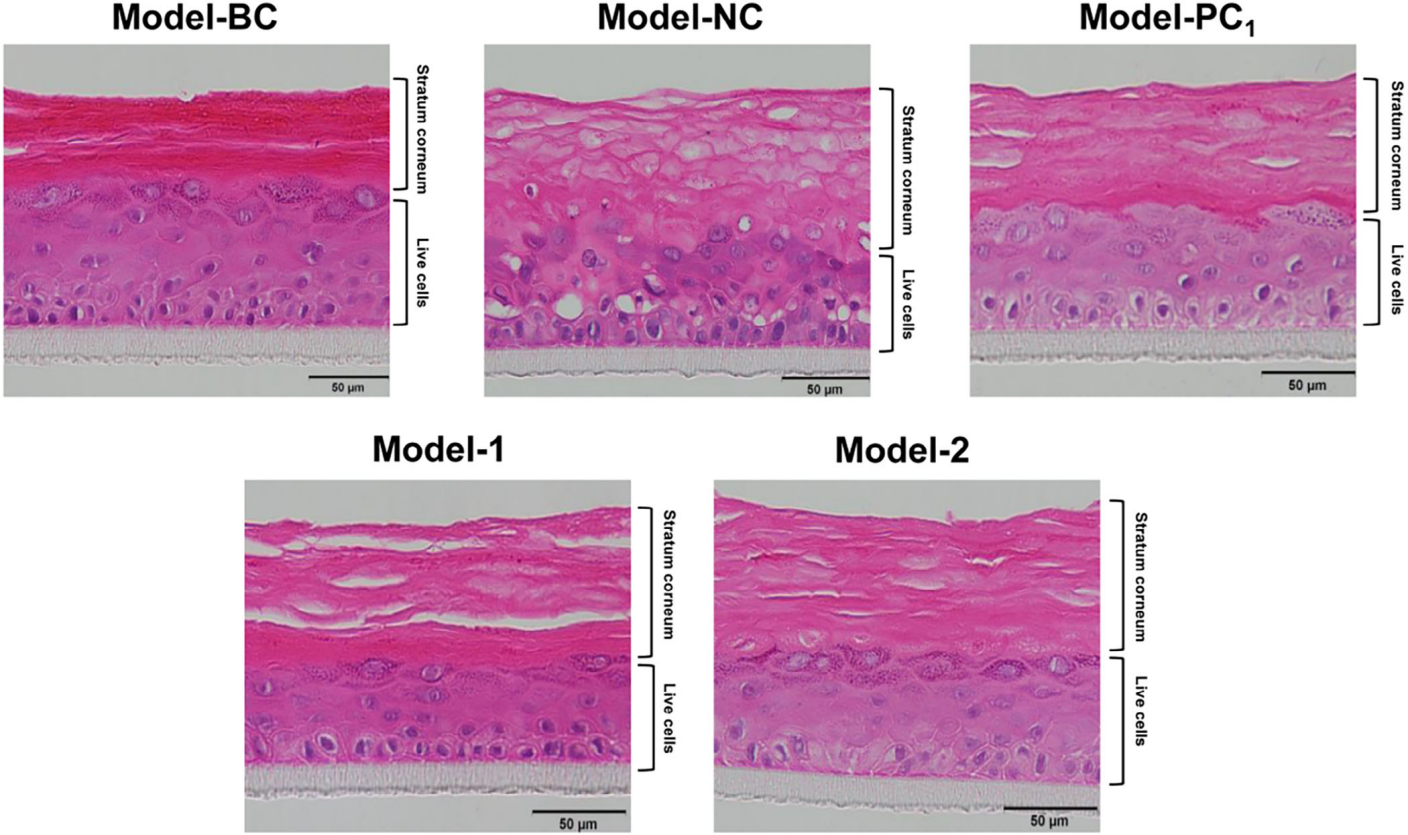
H&E staining results of 3D skin model tests. (Pink color: cytoplasm of cells; blue‐purple color: nuclei) Model‐BC: blank control, Model‐NC: negative control, Model‐PC_1_: 16.19 μg/mL Pirinixic acid, Model‐PC_2_: 100 μg/mL Dexamethasone, Model‐1: 500 μg/mL HA, Model‐2: 250 μg/mL Graft‐1, 250 μg/mL Graft‐3.

Furthermore, the 3D skin model revealed that HA‐Glu effectively improves the repair and tightening of the stratum corneum. In Model‐BC, the keratinocytes displayed a tightly packed arrangement with minimal vacuoles (Figure 5), as opposed to the significantly porous and loose keratinocytes seen in the negative control (Model‐NC). The difference in appearance was attributed to the SLS treatment, which washed away some of the intercellular lipids and caused structural damage to proteins, resulting in a less compact arrangement of the stratum corneum. Additionally, thickening, loosening, and the presence of numerous vacuoles were observed, along with a reduction in the number of viable cell layers. While both HA and HA‐Glu exhibit anti‐inflammatory effects in response to SLS stimulation, their effects on the compactness of the stratum corneum and the thickness of the living cells are different. Specifically, in the case of HA‐Glu, the stratum corneum exhibits a more tightly arranged structure with well‐defined boundaries and more layers of living cells than in the case of native HA. These observations suggest that HA‐Glu may provide additional benefits beyond the anti‐inflammatory effects of HA in improving the overall health and appearance of the skin.

## 4 DISCUSSION

**FIGURE 6:**
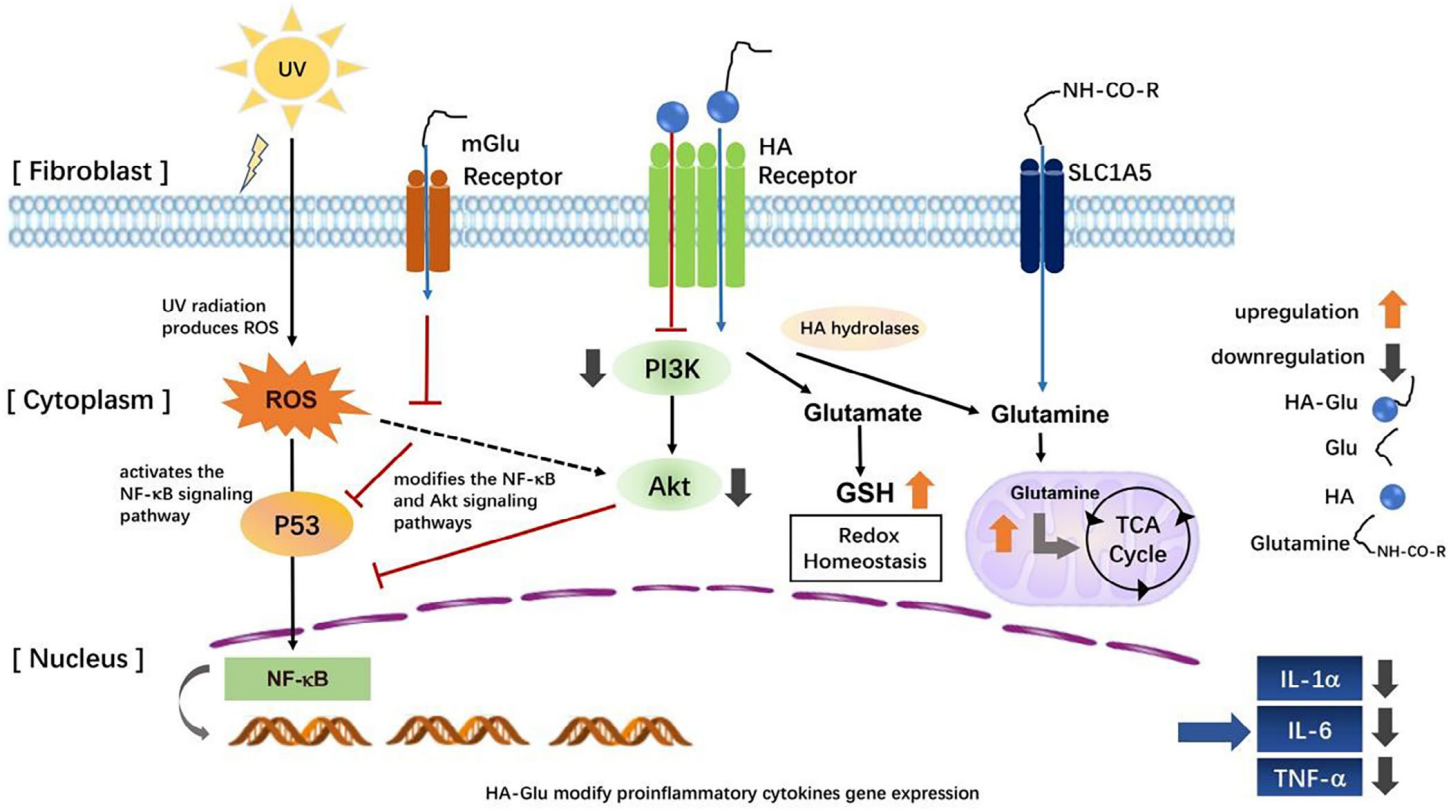
Explanation of the inhibition mechanism of HA‐Glu in HDFs. HDFs, human dermal fibroblasts.

The enhanced functionality of the grafted molecules may result from two aspects: (1) HA intensifies the interaction between Glu and cells; (2) Glu slows down the degradation of HA. The mechanism was illustrated by Figure 6.

Since the grafting degree of HA‐Glu is not 100%, the ungrafted HA segments retain their biological characteristics, such as interaction with receptors such as CD44, RHAMM and HARE, inhibition of PI3K/Akt, regulation of immune responses, and facilitation of ROS scavenging.^18^ This leads to the inhibition of NF‐κB activation, resulting in reduced cytokine release. The engagement between HA segments and CD44 enables HA‐Glu to bind on cell surface, thus enhancing the interaction of Glu with mGlu receptors to regulate cellular inflammatory responses via ROS‐cSrc‐NF‐κB^19^ and Akt^20^ pathways. At the same time, CD44 is involved in cellular uptake of hyaluronan, promoting endocytosis of HA‐Glu into the cell plasma. The internalized HA‐Glu is cleaved by hydrolases within the cell cytoplasm, resulting in the release of glutamate that subsequently generates glutathione (GSH) to maintain redox balance and alleviate oxidative stress.^21^ Hydrolases may also cleave the grafting glutamate site into glutamine, which then migrates into the mitochondria to perform its function. This process includes deamination catalysis by Glutaminase (GLS), followed by the conversion to a TCA cycle intermediate via glutamate dehydrogenase (GDH). This results in ATP synthesis and elevated mitochondrial ATP levels.^21^, ^22^

Additionally, the studies by Carole^16^, ^23^ demonstrated that HA derivatives, such as amino acid derivatives, can effectively decelerate HA degradation. This allows HA to persist in the extracellular matrix and sustain receptor binding for an extended period. Moreover, these derivatives have superior antioxidant properties compared to native HA, thereby inhibiting the activation of NF‐κB signaling.^24^

However, it should be noted that immune regulation in the human skin involves complex cellular interactions, including the roles of Langerhans cells and macrophages, both of which are critical in anti‐inflammatory processes. In addition, the nervous and endocrine systems contribute to the modulation of the immune system. These aspects were not investigated in this work.

## 5 CONCLUSIONS

Cellular experiments and 3D skin model tests were used to reveal the anti‐inflammatory properties of the HA‐Glu molecule. Results indicated that HA‐Glu presents stronger abilities to inhibit inflammation compared to native HA, particularly in regulating IL‐1α, IL‐6, and TNF‐α. Additionally, HA‐Glu effectively promotes the repair of damaged skin by improving the compactness of the stratum corneum and increasing the thickness of the live cell layer. Therefore, HA‐Glu would be effective in alleviating inflammation‐induced skin symptoms and skin aging.

## CONFLICT OF INTEREST STATEMENT

The authors declare no competing financial interest.

## Data Availability

The datasets used and/or analyzed during the current study are available from the corresponding author on reasonable request.
